# Building a network for multicenter, prospective research of central nervous system infections in South America: Process and lessons learned

**DOI:** 10.1016/j.ensci.2018.07.001

**Published:** 2018-07-04

**Authors:** Christina Nelson, Nicanor Mori, Thanh Ton, Joseph Zunt, The Meningoencephalitis Working Group, T. Kochel, A. Romero, N. Gadea, D. Tilley, E. Ticona, J. Soria, V. Celis, D. Huanca, A. Delgado, M. Rivas, M. Stiglich, M. Sihuincha, G. Donayre, J. Celis, R. Romero, N. Tam, M. Tipismana, I. Espinoza, M. Rozas, A. Peralta, E. Sanchez, L. Vasquez, P. Muñoz, G. Ramirez, I. Reyes

**Affiliations:** a Fogarty International Center, National Institutes of Health, Bethesda, MD, USA; b Division of Vector-Borne Diseases, National Center for Emerging and Zoonotic Infectious Diseases, Centers for Disease Control and Prevention, Fort Collins, Colorado, USA; c US Naval Medical Research Unit-6, Callao, Peru; d Department of Neurology, School of Medicine, University of Washington, Seattle, WA, USA; e Departments of Global Health and Medicine, School of Medicine, University of Washington, Seattle, WA, USA; f Dos de Mayo Hospital, Lima, Peru; g Hospital Belen, Trujillo, Peru; h Hospital Edgardo Rebagliati Martins, Lima, Peru; i Hospital San Bartolome, Lima, Peru; j Hospital Cesar Garayar García, Loreto, Peru; k Hospital Felipe Santiago Arriola Iglesias, Loreto, Peru; l Hospital Daniel Alcides Carrion, Callao, Peru; m Hospital Cayetano Heredia, Lima, Peru; n Hospital Regional Cusco, Cusco, Peru; o Hospital Carlos Alberto Seguin, Arequipa, Peru; p Hospital Hipolito Unanue, Lima, Peru; q Hospital Emergencias Pediatricas, Lima, Peru

**Keywords:** Central nervous system infections, Encephalitis, Epidemiology, Herpes simplex virus, Virology

## Abstract

Multicenter collaborative networks are essential for advancing research and improving clinical care for a variety of conditions. Research networks are particularly important for central nervous system infections, which remain difficult to study due to their sporadic occurrence and requirement for collection and testing of cerebrospinal fluid. Establishment of long-term research networks in resource-limited areas also facilitates diagnostic capacity building, surveillance for emerging pathogens, and provision of appropriate treatment where needed. We review our experience developing a research network for encephalitis among twelve hospitals in five Peruvian cities since 2009. We provide practical suggestions to aid other groups interested in advancing research on central nervous system infections in resource-limited areas.

## Highlights

• This report provides a comprehensive review of implementing a research network at twelve hospitals in five Peruvian cities.
• The network helped build testing capacity, provide education about clinical procedures, and gather data about CNS infections.
• Lessons learned offer guidance to others wishing to build similar networks for CNS infections or other unrelated disorders.

## 1 Introduction

Encephalitis is an inflammation of the brain cortex caused by infectious and non-infectious conditions. Encephalitis due to viral infection is usually sporadic, and the causative pathogen typically remains unknown for the majority of patients affected [1]. Although viral encephalitis is typically associated with high morbidity and mortality, effective treatment is available for certain pathogens including herpes simplex virus (HSV) [2].

Given ever-increasing globalization, ongoing research and surveillance of central nervous system (CNS) infections is essential for the identification of emerging and re-emerging pathogens. Although outbreak reports and surveillance for individual pathogens provide valuable information, these approaches do not offer a comprehensive picture of the many potential infectious etiologies causing encephalitis and other CNS infections. Furthermore, nearly all prospective multicenter studies of encephalitis have been conducted in resource-rich countries, such as France, England, Finland, and the United States [1,[3], [4], [5]]. In resource-limited countries, prospective studies have been performed at individual referral hospitals [6,7], but to our knowledge only one multicenter, prospective study of encephalitis conducted in a resource-limited country has been published [8].

Conducting prospective research in a variety of geographic areas permits identification of distinct pathogens prevalent in each region, temporal trends, and risk factors for acquiring these infections. Moreover, research networks can enhance local health care practice, enable diagnostic capacity building, and facilitate improved communication between local and national governmental organizations to advise where specific treatments are most needed. However, published information about the process of building and implementing an encephalitis research network is limited.

We established a multicenter, hospital-based research network for encephalitis that currently includes twelve hospitals in 5 distinct geographic regions of Peru. Initial results of a prospective study on encephalitis conducted through this network have been published previously [8]. This article reviews the process of building a research network and lessons learned during this experience. These principles and practices could be used to develop similar research or surveillance networks for encephalitis and other sporadic infectious conditions in other resource-limited global settings.

## 2 Methods

### 2.1 Assessing existing gaps and securing research funding

Interviews with 48 neurologists across Peru helped lay the groundwork for the research network. When asked to define research priorities, neurologists consistently identified CNS infections as one of their top priorities. A major need identified was improved diagnostic assays for cerebrospinal fluid (CSF). This preliminary research was funded by an R21 NIH Fogarty Brain Disorders grant and formed the basis for an R01 NIH Fogarty International Center grant, which funded the full research network project.

During the planning stages, review of the scientific literature provided information regarding international surveillance standards and helped guide development of the framework in which the network was initiated. Table 1 summarizes the protocols and pertinent findings of recent prospective, multicenter encephalitis studies.

**Table 1:** Prospective, multicenter studies of encephalitis conducted since 1990.

Study location & years (lead author)	Study population & case definition	Approach to testing	Number of patients enrolled	Major findings
California, 1998–2005 (Glaser) [5]	Hospitalized, immunocompetent patients ≥6 months of age. Encephalopathy (defined as depressed or altered level of consciousness lasting ≥24 h, lethargy, or personality change) plus ≥ 1 of the following: • fever • seizure • focal neurological findings • CSF pleocytosis • EEG or neuroimaging findings consistent with encephalitis	Core testing on all patient samples for 16 pathogens including HSV, enteroviruses, measles, West Nile virus, respiratory viruses, and *Mycoplasma pneumoniae*. Selective testing for additional pathogens based on exposure history, symptoms, or physician request.	1570	Despite extensive testing, a confirmed or probable agent was identified in only 16% of patients. Among those: • 69% were viral – enteroviruses (17% of total patients), HSV-1 (16%), varicella zoster virus (9%) • 20% were bacterial – *Mycobacterium tuberculosis* (8% of total patients), pyogenic bacteria (6%), *Bartonella* species (5%)
England, 2005–2007 (Granerod) [1]	Hospitalized patients of any age. Encephalopathy (defined as altered consciousness lasting ≥24 h, including lethargy, irritability, or a change in personality and behavior) plus ≥ 2 of the following: • fever or history of fever during the presenting illness • seizure • focal neurological findings (with evidence of brain parenchyma involvement) • CSF pleocytosis • EEG or neuroimaging findings consistent with encephalitis	Core testing on all patient samples for common causes of encephalitis, plus additional core testing for immunocompromised patients and those who had traveled abroad. Selective testing for additional pathogens as determined by expert panel review.	203	An infectious agent was identified in 42% of patients. Among those: • 67% were viral – HSV (19% of total patients), varicella zoster virus (5%), enteroviruses (1%) • 30% were bacterial – *Mycobacterium tuberculosis* (5% of total patients), Streptococci (2%), *Neisseria meningitidis* (1%) An immune-mediated cause was identified in an additional 21% of patients.
France, 2007 (Mailles) [3]	Hospitalized patients ≥28 days of age. Encephalitis defined as all of the following: • acute onset of illness • ≥1 abnormality of the CSF (white blood cell count ≥4 cells/mm^3^ or protein level ≥ 40 mg/dL) • temperature ≥ 38 °C • decreased consciousness, seizures, altered mental status, or focal neurologic signs Patients with noninfectious CNS disease were excluded.	Diagnostic testing was performed in three successive steps according to the French Society of Infectious Diseases guidelines. Steps are determined by the frequency of infectious agents as a cause of encephalitis and the need to begin early treatment for some pathogens.	253	An infectious agent was identified in 52% of patients. • 69% were viral – HSV (22% of total patients), varicella zoster virus (8%), cytomegalovirus (1%), Epstein-Barr virus (1%) • 30% were bacterial – *Mycobacterium tuberculosis* (8% of total patients), *Listeria monocytogenes* (5%), *Mycoplasma pneumoniae* (1%), *Borrelia burgdorferi* (1%)
Finland, 1993–1994 (Koskiniemi) [4]	Hospitalized children aged 1 month – 15 years. Acute onset (≤4 weeks) of neurological signs lasting ≥24 h (including depressed consciousness, pareses, sensory symptoms, seizure, etc.)^a^	Core testing on all patient samples for common and less common pathogens.	175	An infectious agent was identified in 63% of patients. Among those: • 94% were viral – varicella zoster virus (14% of total patients), respiratory viruses (13%), enteroviruses (12%), HSV (3%) • 5% were bacterial – *Chlamydia pneumoniae* (3% of total patients), *Mycoplasma pneumoniae* (1%), *Borrelia burgdorferi* (1%)

HSV = herpes simplex virus, EEG = electroencephalography, CSF = cerebrospinal fluid.

a In cases where neurologic signs lasted <24 h, patients were included if they had EEG or CSF findings suggestive of encephalitis or depressed consciousness plus ≥1 additional characteristic neurologic sign.

In addition to scientific publications, we contacted colleagues at national and local levels to identify additional gaps in knowledge, research priorities, and existing diagnostic capacity. Other useful sources of information included abstracts and presentations from conferences and local meetings, pilot data collected by colleagues, and available statistics from the Ministry of Health and other local public health agencies. Reviewing this information helped avoid duplication of efforts and guided development of a list of potential pathogens and diagnostic assays specific to different geographic regions.

### 2.2 Establishing case definitions and goals of the research network

We consulted existing literature to determine a case definition that would allow comparison of results across studies and countries, enable compilation of data for meta-analyses, and permit generalizations across populations and countries. Most case definitions included acute onset of neurologic signs and symptoms, such as altered level of consciousness, lethargy, personality change, or seizures. Many also required patients to have one or more additional signs or symptoms suggestive of encephalitis, such as fever, headache, focal neurologic signs, elevated white cell count (pleocytosis) in the (CSF), neuroimaging findings indicative of encephalitis, or electroencephalogram abnormalities (Table 1) [1,5,9].

Diagnostic assays performed on CSF successfully detect an etiologic agent in <20% of patients with presumptive encephalitis [5]. Therefore, defining cases as confirmed, probable, or possible infection provides useful criteria that reflect level of diagnostic certainty. This in turn permits future sensitivity analyses to provide conservative, semi-conservative and non-conservative estimates of prevalence and incidence. Encephalitis is typically considered confirmed when an infectious agent is identified in the CSF via polymerase chain reaction (PCR) assay or culture. Probable and possible definitions typically require detection of a host immune response by serology or identification of infectious agents in other sterile fluids outside the CNS [1,3,6].

The sensitivity and specificity of the case definition should reflect the goals of the research or surveillance. When the primary goal is to identify outbreaks or new infectious agents, high sensitivity is desirable to avoid missing potential cases, since some patients may not present with classic findings. In contrast, if the primary goal is to provide information that will be used to guide patient management, then highly specific definitions may be preferable to ensure correct treatments are administered. Epidemiologically speaking, since non-systematic disease misclassification can bias results towards the null, a highly specific disease definition can reduce misclassification and help uncover important underlying associations.

### 2.3 Identifying potential collaborators and assessing laboratory capacity

The first step in identifying potential collaborators was to determine which geographic locations would be included in the network. For sporadic infectious diseases such as encephalitis, including a variety of geographically diverse but defined areas allows ascertainment of the majority of cases within a defined population and estimation of population-based incidence rates. Within this framework, testing algorithms may be tailored for infections concentrated in particular geographic environments, such as arboviruses in jungle regions. Consulting existing data sources, clinicians, and local public health personnel helped us identify locations where endemic and sporadic infections were most likely to occur.

The next step was to determine which healthcare workers evaluated and managed patients with CNS infection. Regional variations were common, with some patients managed by neurologists, others by internists, infectious disease specialists, or a combination of health care providers. We met with Ministry of Health representatives (local, regional, and national), hospital directors, local researchers, and clinicians to discuss goals of the network and seek their input and suggestions. Defining potential benefits for both research subjects and institutions was equally important and included covering costs of diagnostic testing and treatment for patients, capacity building, and collaboration in the research and publication of results.

Collaborations were facilitated by existing Memoranda of Understanding between the University of Washington and the Universidad Nacional Mayor de San Marcos, Universidad Peruana Cayetano Heredia and the US Naval Medical Research Unit-6 (NAMRU-6) which supported joint research and training activities. As study performance sites were recruited, discussions with the hospital director and research director (when applicable) were followed by a formal letter of agreement from the director to participate in the study. A physician representative from each study site was responsible for communication with the research network point of contact (N.M.), who was responsible for day-to-day activities associated with the study. The research network principal investigators (J.Z. and Dr. Silvia Montano) led the network and discussed issues via email and Skype when needed and via quarterly in-person meetings.

Laboratory capacity was assessed during the planning stage to determine which laboratories were capable of performing basic CSF chemistries (cell count, glucose, and protein), culture, PCR, immunoglobulin assays, and additional advanced diagnostic testing. We encountered no laboratory that was capable of performing all analyses; most were able to perform routine CSF chemistries and culture, although some relied on private local laboratories to perform these assays. Although our long-term goal is to build capacity to perform CSF PCR testing at regional hospitals, we started with providing training and equipment to process and ship CSF and serum samples to a center reference lab in Lima, where an HSV PCR protocol was initiated via technology transfer facilitated by our group. We are in the process of developing a reference center for CSF diagnostics at the Instituto Nacional de Ciencias Neurologicas – the only reference center for neurologic diseases in Peru; this process is a joint effort between the University of Washington and Universidad Nacional Mayor de San Marcos.

### 2.4 Planning study procedures

When planning the research network, we carefully considered what information was necessary and how it would be collected. As most physicians have very limited time, we hired study personnel from among the hospital staff to help subjects or their representatives complete a questionnaire regarding demographic information, symptoms, and medical history. Research-related tasks were generally performed during additional hours outside of regular duties so as not to impact patient care. The referring physician completed only a brief separate form detailing the clinical history and examination. Brain imaging with computed tomography (CT) or magnetic resonance imaging (MRI) is considered standard of care in Peru but is usually not affordable to patients without health insurance. In our study, we provided brain imaging, when clinically indicated, for patients who did not have health insurance. Table 2 provides a summary of the study procedures offered to enrolled patients.

**Table 2:** Summary of study procedures for central nervous system infections in Peru.

Study phase	Enrollment criteria	Clinical procedures & general laboratory testing	Pathogen-specific diagnostic testing
Phase I – Focus on HSV and a limited number of additional pathogens that cause encephalitis	Patients ≥28 days of age with acute onset (<2 weeks) of neurologic symptoms (change in level of consciousness, seizure, altered coordination, or dysphasia) plus one or more of the following: • fever (temperature ≥ 38 °C) • headache • CSF white blood cell count >5 leukocytes/ml • neuroimaging or EEG abnormalities suggestive of encephalitis	Complete blood count; CSF protein, glucose, cell count & differential; Computed tomography scan or magnetic resonance imaging when indicated. IV acyclovir provided and initiated for all patients with suspected HSV infection. Acyclovir discontinued if HSV test results were negative.	Serology: HSV-1 & 2, HIV, HTLV I & II, *Treponema pallidum* Blood PCR: HSV-1 & 2 CSF PCR: HSV-1 & 2 (results typically reported to provider within 72 h), *Mycobacterium tuberculosis,* *Cryptococcus neoformans.* Arboviral testing for a subset of samples (Alphavirus, Flavivirus, and Bunyavirus). Pharyngeal & rectal swabs: Enterovirus
Phase II – Inclusion of additional pathogens	Same as above	As above, plus MassTag PCR encephalitis panels	CSF PCR: RNA Panel: Nipah, Japanese encephalitis, parechovirus, Powassan, La Crosse, LCMV, St. Louis encephalitis, enteroviruses, West Nile, WEE, VEE, rabies, influenza A DNA Panel: adenoviruses, cytomegalovirus, Epstein-Barr, varicella zoster, HHV-1,2,6, *H. influenzae,* *S. pneumoniae, N. meningitidis,* *L. interrogans, M. tuberculosis, T. gondii, C. albicans, C. neoformans*

HSV = herpes simplex virus, EEG = electroencephalography, CSF = cerebrospinal fluid, HTLV = human T-cell lymphotropic virus, LCMV = lymphocytic choriomeningitis virus, WEE = Western equine encephalitis, VEE = Venezuelan equine encephalitis, HHV = human herpes virus.

While lumbar puncture is considered standard of care for patients with suspected encephalitis – and is therefore not considered an additional study procedure – it is often deferred due to patient anxiety, stigma, or fear the procedure may cause harm. In addition, healthcare providers sometimes feel uncomfortable performing lumbar puncture. To overcome these issues, a neurologist (N.M.) visited each site to evaluate physician comfort with performing lumbar puncture and demonstrate proper technique for those providers who did not feel comfortable performing the procedure. Following these site visits, we noted enrollment at these sites increased and providers who had previously reported that patients refused lumbar puncture found that patients accepted the procedure more readily – perhaps reflecting increased provider confidence with counseling patients and performing the procedure.

Breadth of laboratory testing was tailored to the goals of the research, existing laboratory capacity at each site, and local practices. While some prior studies tested all samples for a predetermined list of pathogens [6], others employed a stepwise or targeted testing approach based on patient risk factors and clinical presentation (Table 1) [3,5]. Studies sometimes included an expert panel that reviewed patient medical records and recommended further testing based on the clinical picture (1). In addition, testing was sometimes performed for non-infectious etiologies (e.g. autoimmune disease) and comorbid conditions such as HIV infection.

### 2.5 Obtaining study approval

Institutional Review Board (IRB) approvals were obtained from the principal investigator's (J.Z.) institution as well as host country IRBs associated with collaborating universities and local participating hospitals. This was a lengthy and complicated process, especially with the multiple sites involved. Some smaller hospitals without IRBs had agreements with local academic institutions that served as the reference IRB; in these situations, a letter of cooperation from the hospital director was required. During the research planning stages, meetings with all study personnel were held to review responsible conduct of research with human subjects.

### 2.6 Building diagnostic and research capacity

**Fig. 1:**
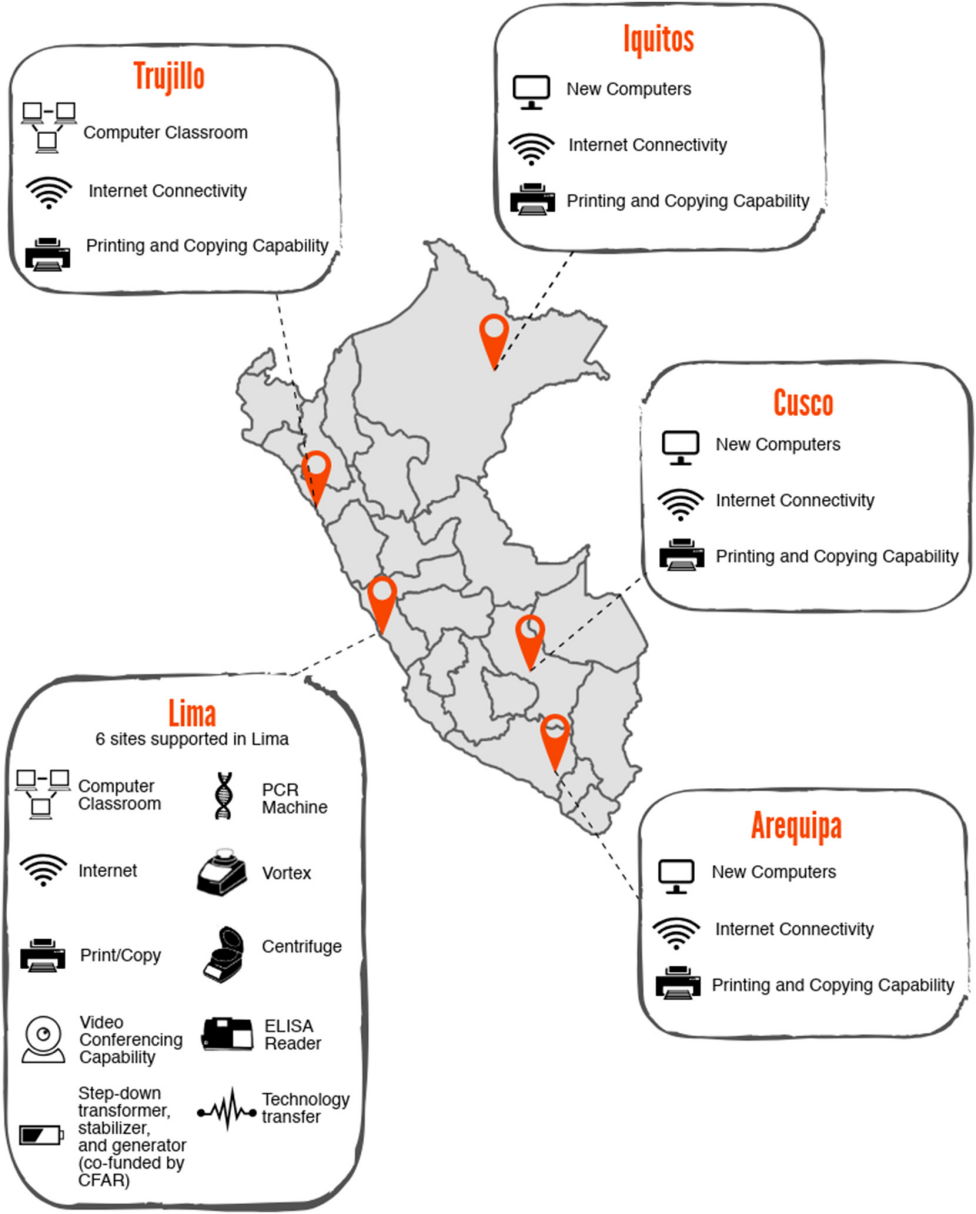
Map of research network sites in Peru and associated capacity building. ELISA = enzyme-linked immunosorbent assay, CFAR = Center for AIDS Research.

Building capacity at study sites was essential to the success and sustainability of research and surveillance, and in many cases was an important aspect of ethical research. Enhancing capacity took a variety of forms (Fig. 1), including:

1) Improving laboratory infrastructure for processing and storing samples (e.g. freezers, incubators) and diagnosing infection (e.g. PCR machines). Plans for maintenance and repair of equipment were also discussed and incorporated into each hospital's system.
2) Augmenting infrastructure for storing data and communicating results (e.g. computers with stable internet connection);
3) Providing training opportunities for study and laboratory personnel in clinical research and good laboratory practices and clinical research; and
4) Increasing research capacity through workshops on research methodology, data management and analysis, and manuscript writing. To this end, we piloted a two-week research methodology course developed jointly by Peruvian and U.S. collaborators and offered via Adobe Connect to collaborators located at remote sites. A certificate of completion was awarded to each participant who attended the workshop.

### 2.7 Initiating research

Once potential collaborators were identified and we had initiated IRB application processes, we began providing courtesy testing of CSF samples from potential study sites (e.g. PCR testing for HSV). While no study data was obtained during this period, this service allowed study personnel to fine-tune protocols for sample processing, transportation, and reporting of test results and strengthen working relationships between collaborators.

**Fig. 2:**
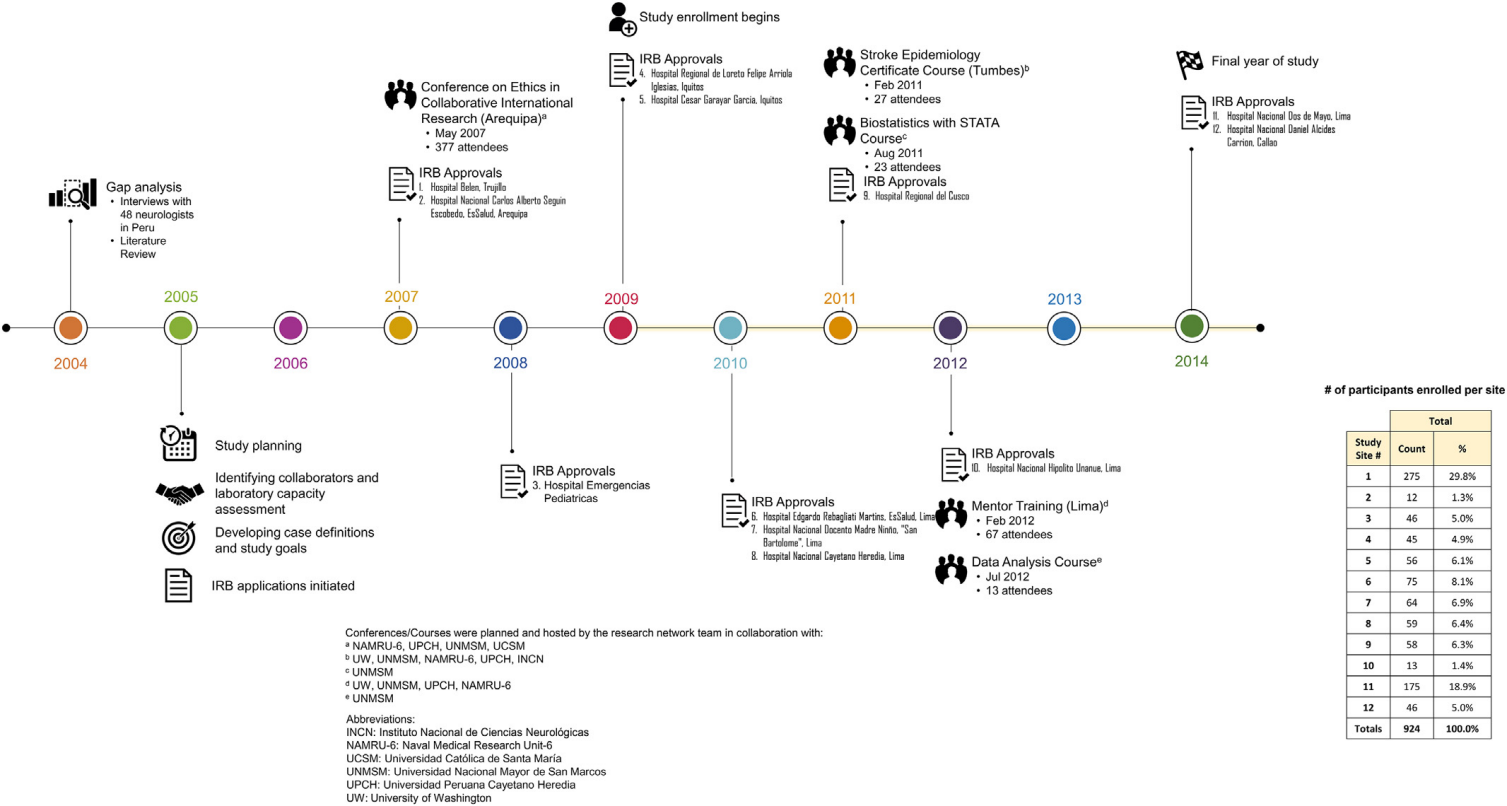
Timeline for building a multicenter, prospective research network on central nervous system infections.

Once IRB approvals were obtained, we simultaneously began enrolling patients in the capital city of Lima and Trujillo, a coastal city in northern Peru (Fig. 2). For each study site, staff members were identified to enroll patients, perform study examinations, and process samples. In our experience, hiring a part-time study coordinator at each study site was extremely helpful to assist with implementing and maintaining the study. A password-protected, web-based data entry form facilitated data input and tracking of test results.

For laboratory diagnosis, we used a spoke and hub model, with basic tests performed at study sites and more complex diagnostics, such as HSV PCR, sent to the US NAMRU-6 in Lima for processing. This model required additional coordination to arrange procurement of dry ice, sample shipment, data entry and tracking, and reporting of results to sites.

### 2.8 Patient benefits – providing intravenous acyclovir and reporting test results

As intravenous (IV) acyclovir is the only medication recommended for treatment of HSV encephalitis, and without treatment 70% of patients with HSV encephalitis would die, we decided – in collaboration with Peruvian colleagues – that we were ethically obligated to provide IV acyclovir to patients with presumptive HSV encephalitis enrolled in the study. Acyclovir was initiated in all subjects with suspected HSV encephalitis and continued for 14 days if the HSV PCR was positive, or discontinued if negative. As part of the study protocol, health care providers were given information about the benefits and risks (e.g. crystal-induced nephropathy) of acyclovir.

Unfortunately, IV acyclovir was not widely available in Peru. We initially purchased bulk IV acyclovir through a US hospital pharmacy, but a national shortage led to a search for other potential suppliers. Certain challenges arose in the acyclovir supply chain, including shipping and manufacturing delays.

Timely reporting of test results to clinicians was vital to guide treatment and patient care. In our research network, HSV PCR results were typically reported within 72 h. This was the most clinically important test since this determined whether IV acyclovir treatment was continued. Although we were determined to provide test results to providers in a timely manner, we were careful to inform investigators that delays were possible as delays in shipping and processing did occur.

In addition to diagnostic testing and IV acyclovir, lumbar puncture kits were also provided to study hospitals and the cost of brain imaging studies (computed tomography or magnetic resonance imaging) was covered when indicated for patients without insurance.

### 2.9 Management and expansion of the research network

Research involving multiple sites offers many advantages, including increased power for identifying rare diseases, decreased bias related to site or region-specific selection factors, and new opportunities for future multi-site research collaborations. As the speed of IRB approval at different sites was variable, we gradually increased the network size over the first few years (Fig. 2). Efforts to increase the size of the surveillance network were aided through presentation of initial study findings at national meetings, word of mouth, and communication with medical and public health associations in Peru.

As research progressed, we periodically analyzed study data to ensure accuracy and identify opportunities for improvement. Evaluations included reviewing demographics, recruitment patterns, and preliminary test results to identify suboptimal patient recruitment at specific sites, protocol deviations, and other issues that required correction. Regional or seasonal outbreaks of specific or unexpected pathogens might also be detected during ongoing review and should be reported in a timely manner.

Regular site visits were important for assessing recruitment and laboratory processes and soliciting input from collaborators about what was working well and what areas needed attention. Periodic laboratory proficiency testing was initiated to ensure laboratory testing errors were recognized and corrected; this process also informed development of laboratory capacity at new sites.

Over time, physicians outside of the network heard about this project and expressed interest in receiving assistance with diagnostic testing and joining the network. We provided testing of samples as a courtesy, without collecting patient information for the study, and in some cases built collaborations that led to addition of new study sites. Prior to addition as a new study site, study personnel visited the potential site to explain the study and answer questions. IRB modifications were required for each site addition.

Laboratory testing via the network was expanded to include additional pathogens based on local needs. After implementing testing for viral encephalitis, we were approached by pediatricians who expressed interest in adding bacterial testing to the study protocol. In addition, to provide a more complete understanding of the importance of enteroviruses as an etiology of encephalitis in Peru, we added testing for enterovirus on CSF samples in which no pathogen had been detected on initial testing. Addition of new assays required additional training of laboratory personnel, purchase of additional laboratory materials, and modification of the IRB.

Finally, nested studies within the surveillance program provided opportunities for investigators to conduct research with little extra logistical change or expense. For example, one research fellow (C.N.) added a nested case-control study to examine risk factors for HSV encephalitis. Patients enrolled in the study who tested positive for HSV encephalitis were considered cases, while controls were patients who presented to the respective study hospitals with minor trauma.

## 3 Future directions

Study results were shared with referring physicians, study site staff, and the larger public health community to encourage integration of findings into practice and ultimately policy change. Laboratory testing revealed a substantial proportion of patients with HSV infection across the study sites [8], supporting the need for access to IV acyclovir nationwide. We are currently working with Peruvian officials to explore possibilities for obtaining and maintaining a supply of IV acyclovir in Peru.

Sustainability of new diagnostic and treatment capacity requires investment and participation from regional and local governments. We continue to collaborate with regional government and academic centers to increase laboratory capacity via training laboratory technicians, installing of basic equipment (e.g. for CSF cell count and protein testing), and implementing higher technology diagnostics (e.g. PCR assay). We have also leveraged these improvements with other research and surveillance programs. For example, in Cusco we are working with the Ministry of Health to expand laboratory capacity in collaboration with a separate surveillance study of respiratory infections.

## 4 Conclusions

Research networks are valuable for defining the etiologies of encephalitis and many other conditions, identifying geographic differences in disease patterns, and building capacity. By integrating collaborators across multiple sites and large geographic areas, our research network provided a source of population-based cases for epidemiologic studies. Standardizing research protocols and case definitions across distinct areas of the world would allow more generalizable study findings and lead to a better understanding of the etiologies and epidemiology of encephalitis.

While there are many challenges involved in building a research network, we found that a stepwise progression from assessing existing research infrastructure to establishing a collaborative network enabled successful implementation. Local healthcare providers familiar with local endemic infections, patient referral patterns, and existing capacity were involved during the early planning stages of the network to ensure research was appropriate to each environment and addressed the capacity-building needs of each site. Overall, the surveillance network led to improved healthcare for patients and new research opportunities for collaborating partners and institutions. As the network continues to grow, we anticipate ongoing development of diagnostic and research capacity at regional hospitals and academic centers to ensure sustainability of our joint efforts.

## Competing interest statement

The authors have no conflicts of interest to disclose.
